# Low temperature atomic layer deposition of cobalt using dicobalt hexacarbonyl-1-heptyne as precursor

**DOI:** 10.3762/bjnano.14.78

**Published:** 2023-09-15

**Authors:** Mathias Franz, Mahnaz Safian Jouzdani, Lysann Kaßner, Marcus Daniel, Frank Stahr, Stefan E Schulz

**Affiliations:** 1 Fraunhofer-Institute for Electronic Nano Systems ENAS, Technologie-Campus 3, 09126 Chemnitz, Germany; 2 Chemnitz University of Technology, Straße der Nationen 62, 09111 Chemnitz, Germany; 3 scia Systems GmbH, Clemens-Winkler-Str. 6c, 09116 Chemnitz, Germany; 4 FAP Forschungs- und Applikationslabor Plasmatechnik GmbH, Gostritzer Str. 67B, 01217 Dresden, Germany

**Keywords:** atomic layer deposition (ALD), cobalt, low-temperature ALD, PEALD, plasma-enhanced ALD, XPS

## Abstract

In this work, we present the development of an atomic layer deposition (ALD) process for metallic cobalt. The process operates at low temperatures using dicobalt hexacarbonyl-1-heptyne [Co_2_(CO)_6_HC≡CC_5_H_11_] and hydrogen plasma. For this precursor an ALD window in the temperature range between 50 and 110 °C was determined with a constant deposition rate of approximately 0.1 Å/cycle. The upper limit of the ALD window is defined by the onset of the decomposition of the precursor. In our case, decomposition occurs at temperatures of 125 °C and above, resulting in a film growth in chemical vapour deposition mode. The lower limit of the ALD window is around 35 °C, where the reduction of the precursor is incomplete. The saturation behaviour of the process was investigated. X-ray photoelectron spectroscopy measurements could show that the deposited cobalt is in the metallic state. The finally established process in ALD mode shows a homogeneous coating at the wafer level.

## Introduction

The atomic layer deposition (ALD) of cobalt films is an ongoing topic of interest [1]. Cobalt thin and ultrathin films play an important role in current generations of integrated circuits [2]. Compared to copper, the metal offers a greater resistance to electromigration and lowers the tendency to undergo diffusion, giving a higher stability in environments involving both elevated temperature and high current densities [1,3]. In current technology nodes with device dimensions below 10 nm, electron scattering becomes the dominant factor in copper-based local interconnects. In consequence, other metals such as tungsten and cobalt are used to replace copper. Recent studies show a line resistance benefit of cobalt compared to tungsten [2–5]. Because of its ferromagnetism, cobalt is a frequently used metal for magnetic sensor systems. Typically, these systems require ultrathin layers within the nanometre scale [6].

The thickness and conformality criteria of future microelectronics devices require the development of cobalt metal films deposited by ALD. Because of the self-limiting growth process, ALD allows for the sub-nanometre control of layer thickness while achieving better conformal coatings than any other deposition technique. The commonly used ALD database “Atomic Limits” [7] (based on the reviews of Puurunen [8], Miikkulainen et al. [9], Knoops et al. [10], and Mackus et al. [11]) currently lists 23 different ALD processes for the deposition of metallic cobalt including twelve plasma-assisted processes. Generally, these plasma-assisted ALD processes are reported to be carried out at temperatures above 100 °C. These processes use precursors such as CoCp_2_, Co(EtCp)_2_, or CpCo(CO)_2_ [12–14]. The commonly used precursor dicobalt hexacarbonyl *tert*-butylacetylene (CCTBA) can be used to deposit metallic cobalt in the temperature range from 125 to 200 °C [15]. As an exception, Kim et al. have reported the ALD of Co with Co_2_(CO)_8_ in the temperature range of 70 to 110 °C. However, this process resulted in a significant carbon contamination [16]. Thermal ALD processes operate usually at temperatures higher than 150 °C [17–21].

Characteristic for ALD processes, the growth rate is mainly independent of the substrate temperature in a specific temperature range, often denominated as ALD window. Within this range, the deposition is determined by the self-limiting behaviour of surface adsorption, and the reaction is completed in the second half cycle with an additional reactant. Therefore, the growth rate is nearly independent of the cycle time. The upper limit for this self-limiting growth is usually the thermal decomposition of one of the precursors. In this case, the process is within the regime of chemical vapour deposition (CVD), resulting in a continuous film growth. It is therefore essential to carry out the ALD process in a way that the first precursor only reacts with the second precursor during the second half cycle [22].

Within this study, we present the atomic layer deposition of metallic cobalt using dicobalt hexacarbonyl-1-heptyne [Co_2_(CO)_6_HC≡CC_5_H_11_] as precursor [23]. We show that the deposition can be carried out at low temperatures in the range from 50 to 110 °C by utilizing H_2_ plasma as reducing agent with a deposition rate of approximately 0.1 Å/cycle. The deposited films were analysed by X-ray photoelectron spectroscopy and are well in the metallic state. We also show the optimization of the overall process through varying the pulse times for precursor, purging, and the plasma pulse.

## Experimental

### Process equipment

**Figure 1:**
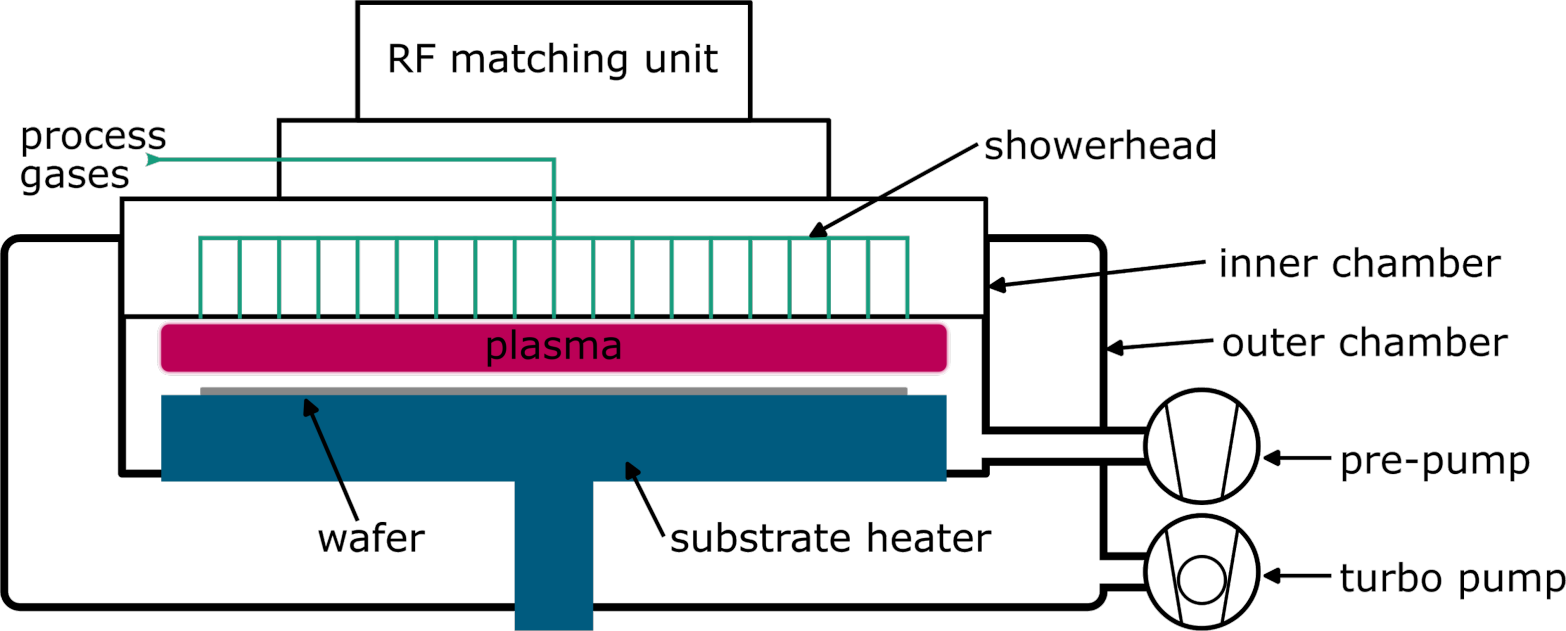
Schematic sketch of the scia Atol 200 processing tool.

The ALD process development was done on a scial Atol 200 single-wafer reactor equipped with cassette loading and a handling robot. This machine was designed and fabricated by scia Systems GmbH in corporation with Fraunhofer Institute for Electronic Nano Systems ENAS, Center for Microelectronics of Chemnitz University of Technology, and FAP Forschungs- und Applikationslabor Plasmatechnik GmbH. The reactor is a single-wafer reactor for 200 mm wafers and consists of an inner and an outer reactor with a typical base pressure of 3 × 10^−6^ mbar. A simplified schematic sketch is shown in Figure 1. The reactor is designed to offer a wide variability as it consists of two bubblers, two Vapbox evaporators (Kemstream SAS), and two CEM systems *(*Bronkhorst High-Tech B.V.) for precursor delivery. In that way, one can evaporate liquids with high and low vapour pressure, as well as solids in solution. The attached RF generator operates at 13.56 MHz, creating a direct capacitively coupled plasma, if required. The system includes an integrated iSE spectroscopic ellipsometer of J.A. Woollam Co. for inline thickness measurements.

### Basic materials and procedure

The whole study was done using single crystal 200 mm silicon (100) wafers with a pre-coated thermal SiO_2_ film of 100 nm thickness. As precursor for all depositions dicobalt hexacarbonyl-1-heptyne [Co_2_(CO)_6_HC≡CC_5_H_11_] was used. The precursor was synthesised according to Georgi et al. [23] and filled to a common 200 mL stainless steel bubbler under inert gas atmosphere. The bubbler was heated to 30 °C, which will result in a vapour pressure of 15.7 mbar according to the published Antoine parameters of Georgi and co-workers [23]. Pure argon (6N) was used as carrier gas for bubbling. The depositions in CVD mode were done with a continuous cobalt precursor delivery and without any further reacting gases. The precursor was provided via the showerhead over the whole wafer surface. The depositions were done at 90, 100, 125, and 150 °C.

The ALD depositions were carried out with molecular hydrogen (H_2_) as second precursor. During the H_2_ pulsing step a direct CCP plasma (50 W) was created for the entire pulsing time. The ALD process consists of cycles with the following pattern: cobalt precursor dosing – argon purging – H_2_ plasma – argon purging. Typical pulse times for this pattern were 6, 1, 2, and 1 s, respectively. For pulse times variations, this pattern has been maintained, except for the altered duration of the cycle step under analysis. Depositions in ALD mode were done at temperatures in the range from 35 to 125 °C.

### Characterisation

The film thickness was measured with the integrated iSE ellipsometer of J.A. Woollam Co. at an angle of 70° towards the wafer normal. However, for measurements the inner reactor has to be opened and the substrate has to be moved to a defined measurement position. This was done after a subset of typically 100 ALD cycles. The film thickness after a series of depositions was determined ex situ using a Sentech SE850 ellipsometer of Sentech Instruments GmbH under the same angle of incidence. A map of 50 measurement points with a spiral pattern was used to determine the film thickness distribution. Both systems used the same model for thickness determination, namely a Drude–Lorentzian model for metallic cobalt according to Ward [24] combined with a Tauc–Lorentzian model for possible occurrences of oxidised cobalt [25–26].

The film compositions were measured ex situ by X-ray photoelectron spectroscopy (XPS) on a PRECAV sp. Z. o. o. XPS system using a MX-650 Al X-ray source and a R3000 analyser from VG Scienta using a fixed pass energy of 200 eV. The sample was pre-cleaned by argon sputtering for 2 min with 4.0 keV acceleration energy to remove surface adsorbents and contaminations. The data were analysed using MATPLOTLIB [27–28] and LMFIT [29]. The XPS spectra were corrected using the common Shirley background [30]. The peaks were fitted with the common Voigt profile. In cases of metallic transitions, a non-symmetric profile was used. The normalised deviation and Abbe criteria were calculated according to Hesse and co-workers [31].

## Results and Discussion

### Exploring the transition from CVD to ALD: determining the upper temperature limit

**Figure 2:**
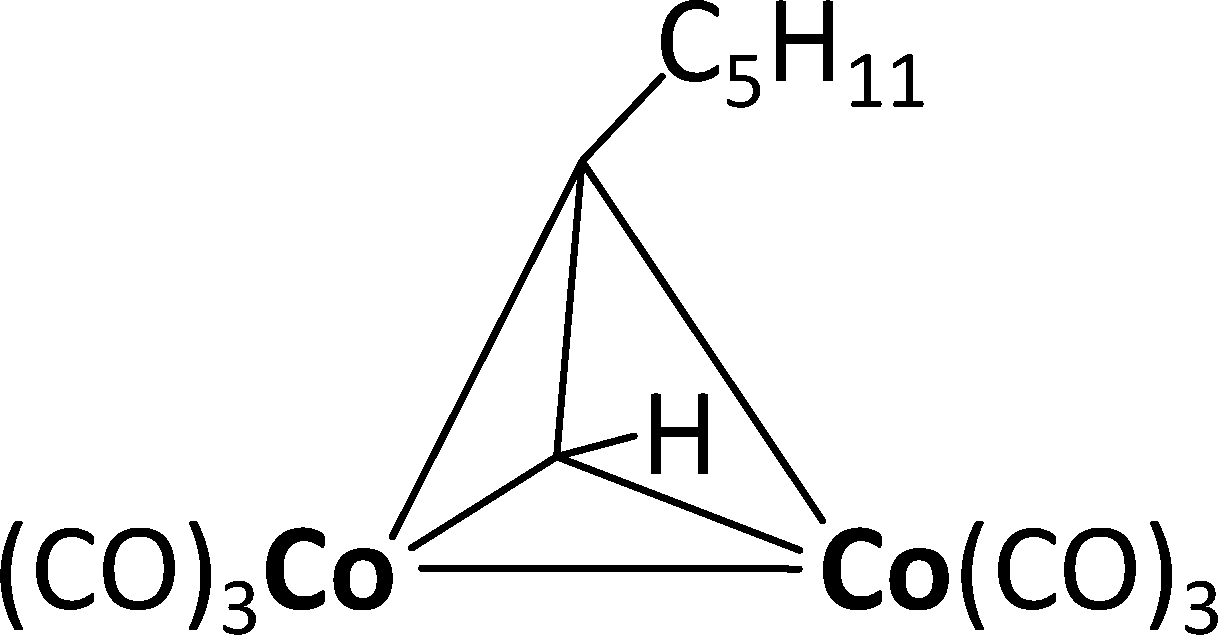
Schematic sketch of the structure of the precursor [Co_2_(CO)_6_HC≡CC_5_H_11_].

The metallic cobalt deposition process is based on the precursor dicobalt hexacarbonyl-1-heptyne [Co_2_(CO)_6_HC≡CC_5_H_11_]. The synthesis and basic characteristics of which have been described by Georgi and co-workers [23]. A schematic sketch of the precursor structure is shown in Figure 2. This precursor was selected because of the following considerations: Georgi et al. provided a set of nine similar complexes with different alkynes and reported their film compositions after CVD experiments. We selected a liquid precursor without Si contaminations and with the lowest amount of remaining oxygen contamination.

In order to determine an upper limit of the ALD window of the chosen precursor, a number of CVD experiments were performed at different temperatures using only the cobalt precursor without further reactants. The aim was to find a temperature where the cobalt precursor does not decompose. In that way, the upper limit of a possible ALD window can be approximated.

**Figure 3:**
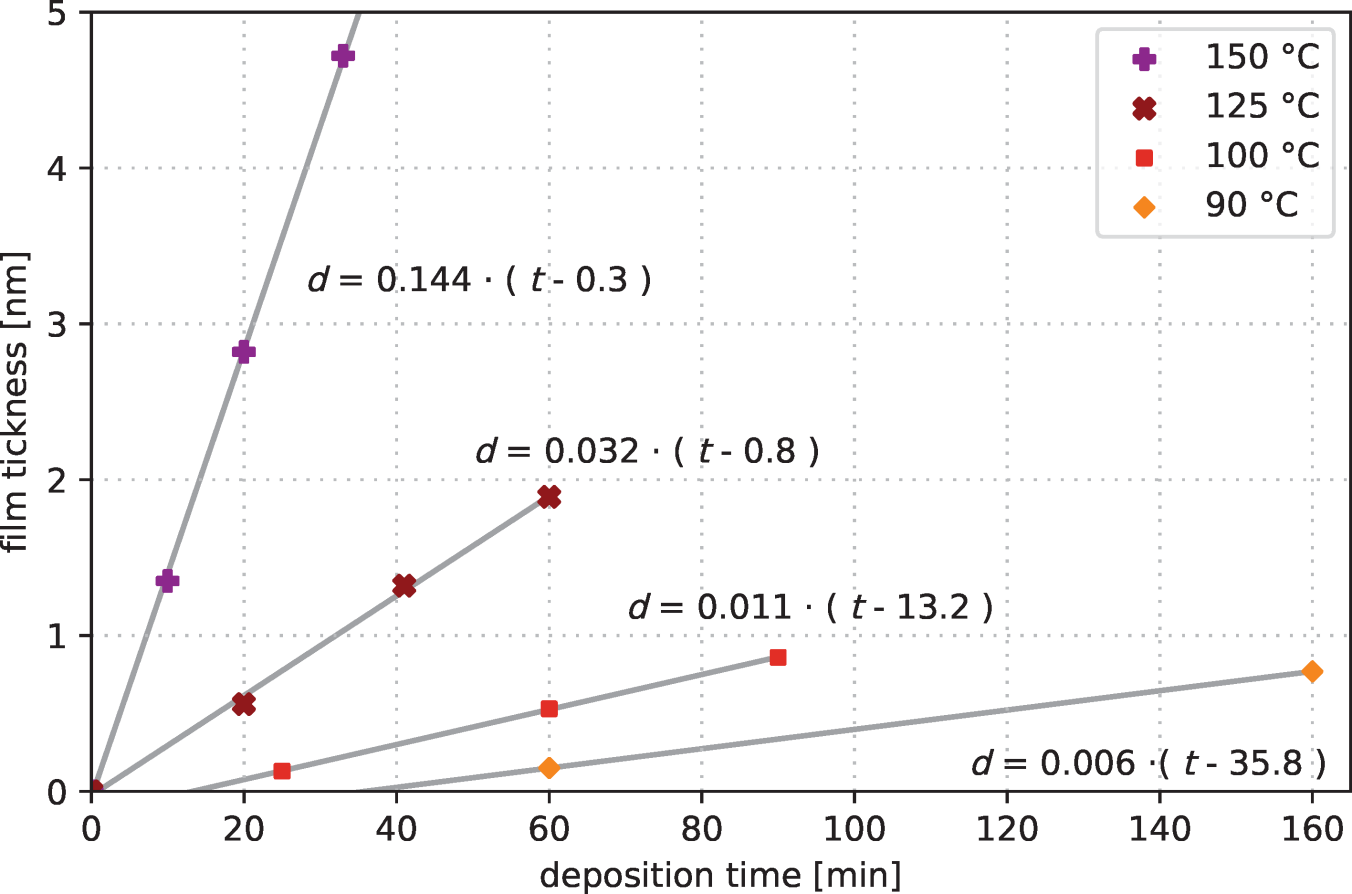
Influence of deposition temperature on film growth rate of continuous CVD depositions. The film growth is based only on thermal decomposition of the precursor.

**Figure 4:**
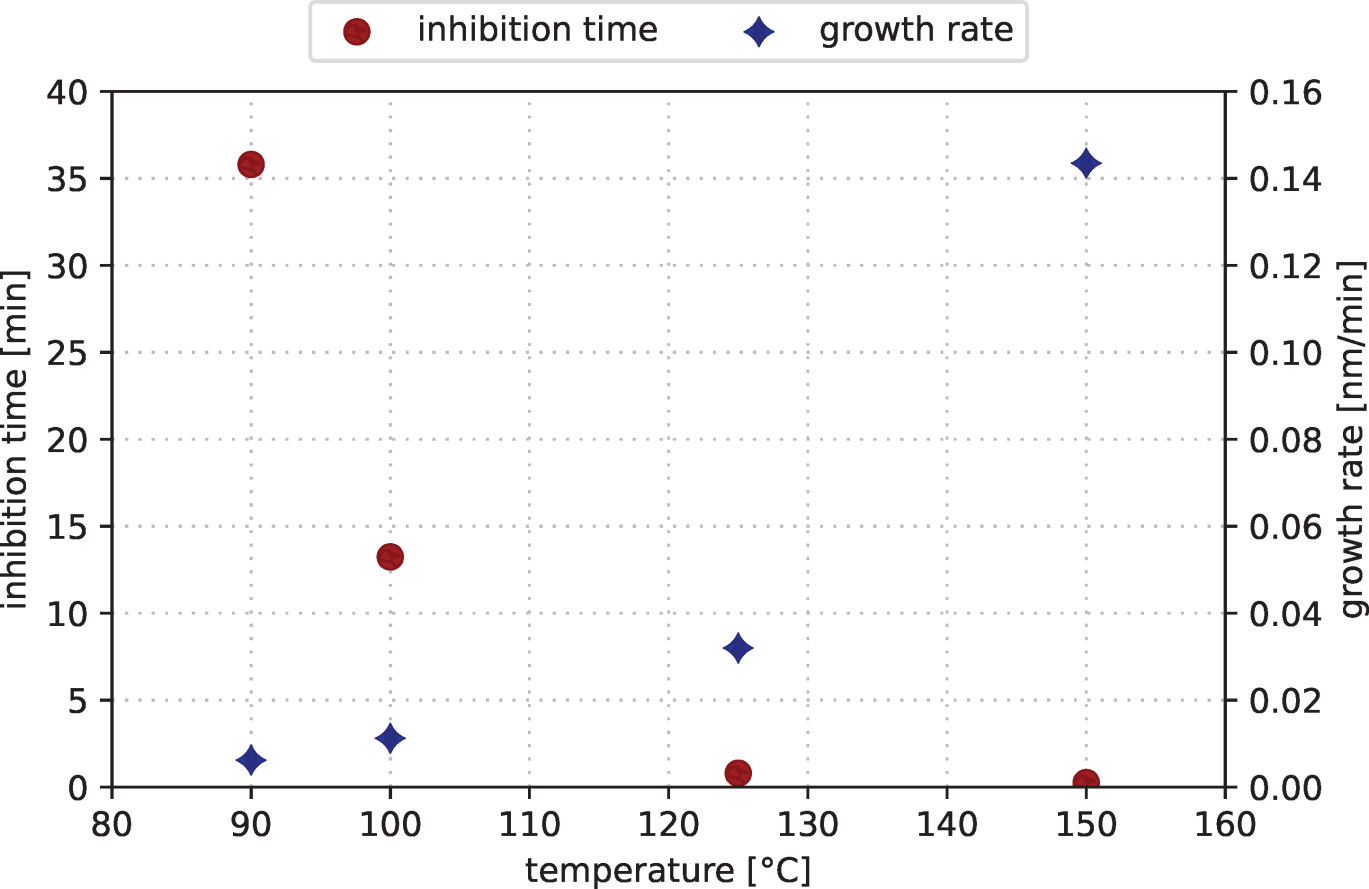
Temperature dependence of inhibition time (red dots) and CVD growth rate (blue diamonds) extracted by using a linear growth approximation.

The CVD experiments of Melzer et al. [32] demonstrated a precursor reaction with O_2_ in the temperature region from 130 to 250 °C to form cobalt oxide. The deposition experiments by Georgi et al. showed a CVD-based layer formation of metallic cobalt at 250 °C with the cobalt precursor [23]. Based on these previous results, the initial deposition temperature for cobalt metal CVD was set to 150 °C and was decreased successively for further processes. Figure 3 shows the film growth trend of these CVD experiments at 150, 125, 100, and 90 °C. The films grow with linear rates. We assume a linear dependency of type *d* = *r*·(*t* − *t*_0_) with film thickness *d*, deposition rate *r*, deposition time *t*, and the inhibition time *t*_0_, that is, the time where no CVD-like growth may occur. At elevated temperatures of at least 125 °C, the linear fits intersect the origin, as one would expect of a continuous decomposition of the precursor on the wafer surface. We assume a common CVD-like growth at temperatures of 125 °C and above. The results at lower temperatures of 100 and 90 °C show that the deposition mechanism has changed. The thickness trend shows that the deposition seems to be inhibited in the initial phase as *t*_0_ rises significantly above 0. The assumed linear relationship has been plotted in Figure 3 for each deposition temperature. The calculated inhibition times are 13.2 min for 100 °C and 35.8 min for 90 °C. The slopes of the linear dependency on the temperature are plotted in Figure 4. This plot also includes the calculated inhibition times according to the assumed linear fit. This simplified assumption shows that the inhibition time in CVD mode rises significantly at temperatures below 125 °C while operating at low deposition rates. This indicates an upper limit for an ALD process window with [Co_2_(CO)_6_HC≡CC_5_H_11_] as precursor around this temperature.

**Figure 5:**
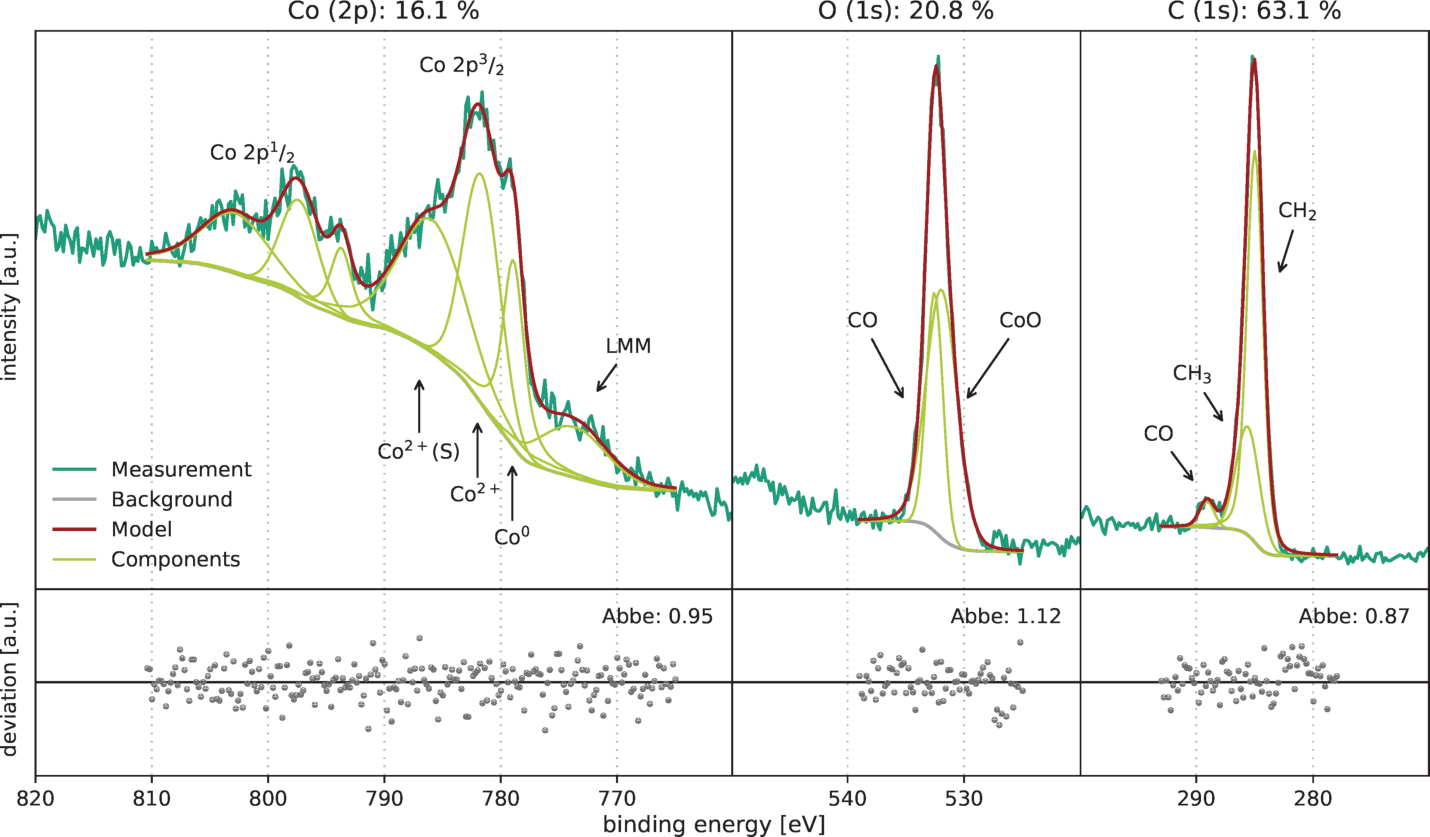
XPS measurements of the CVD film deposited at 150 °C.

The deposited CVD films were analysed with XPS. Figure 5 shows the details of the XP spectra of the film deposited at 150 °C. The overview spectrum is given in Supporting Information File 1, Figure S1. The film mainly consists of the three elements carbon (63.1 atom %), oxygen (20.8 atom %), and cobalt (16.1 atom %).

The main carbon 1s feature is located at 285.0 eV binding energy (Figure 5). This is the typical value for carbon in alkyls [33]. It is likely that this correlates to the carbon bonded as −CH_2_− within the *n*-heptyne group of the used precursor. The second peak at 285.6 eV matches the bonding state of the terminating −CH_3_ groups. The third peak at 289.1 eV is likely correlated to −C=O bonds, especially originating from the carbonyl groups [33]. No evidence could be found of cobalt carbide formation, which would result in binding energies of approximately 284 eV or below [34–35].

The oxygen 1s peak has its maximum at 532.2 eV (Figure 5). However, assuming just one feature will result in a poor fitting result with an Abbe parameter of 0.41, indicating a significant systematic error (see Supporting Information File 1, Figure S2). It is therefore reasonable to assume at least two oxygen components. One is located at 531.9 eV correlated to oxygen in cobalt oxide [36–37]. The higher bonding energy at 532.5 eV correlates to a C=O bonding according to the results from the carbon spectrum [33].

The cobalt 2p peak is split into two parts, the 2p^3^/_2_ and the 2p^1^/_2_ component, because of the spin–orbit coupling. Cobalt in the metallic state (Co^0^) has a 2p^3^/_2_ peak at 777.3 eV [38] or 778.5 eV [39]. The XPS emission lines of oxidised cobalt CoO consist of a core level peak (Co^2+^) and a shake-up satellite (Co^2+^ (S)) [37]. The measured Co 2p^3^/_2_ peak consists of three features (Figure 5). This indicates the presence of metallic as well as oxidised cobalt (mainly Co^2+^). After fitting, the peak with a binding energy of 778.9 eV can be assigned to cobalt (Co^0^) in metallic state. The peaks located at 781.6 and 785.9 eV correlate to oxidised cobalt. The present cobalt is mainly in an oxidised state with just a slight amount of metallic cobalt after deposition in CVD mode at 150 °C.

The precursor [Co_2_(CO)_6_HC≡CC_5_H_11_] consists of 9.5 atom % Co, 28.6 atom % O, and 61.9 atom % C, when ignoring H, which is not measurable in XPS. The measured film composition indicates that the carbonyl groups (−CO) have been partially evaporated, while the heptyne group has been incorporated within the film to some extent.

### ALD with [Co_2_(CO)_6_HC≡CC_5_H_11_] and H_2_ plasma

The CVD experiments showed that the window for temperature-independent depositions in ALD mode can be expected at temperatures below 125 °C, as it was assumed from the temperature-dependent growth rate measurements (Figure 3). We performed a set of deposition experiments for various temperatures in the temperature range of 35 to 125 °C. For all experiments, we used a pattern of 6 s precursor dose, 1 s argon purge, 2 s H_2_ plasma pulse, and 1 s argon purge, for each cycle.

**Figure 6:**
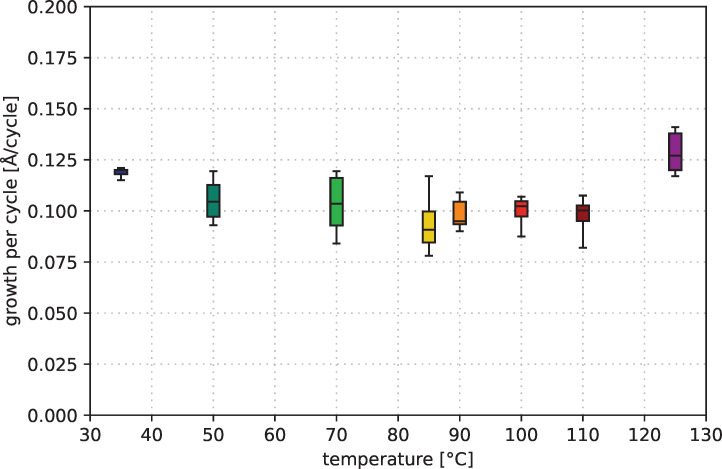
Growth per cycle of ALD processes with [Co_2_(CO)_6_HC≡CC_5_H_11_] precursor at various temperatures.

The temperature dependence of the growth rate for the performed ALD processes is shown in Figure 6. In the temperature region between 50 and 110 °C the deposition rate is almost constant at 0.1 Å/cycle. This deposition rate is lower than that of processes with CCTBA, where a rate of 0.8 Å/cycle could be achieved [15]. The growth rate at 125 °C is significantly higher, indicating a deposition in CVD mode. This is in good agreement with the preliminary results of depositions in CVD mode. The upper limit for ALD depositions is between 110 and 125 °C. The deposition at 35 °C also shows an increased growth rate indicating a lower limit of the ALD window between 35 and 50 °C.

**Figure 7:**
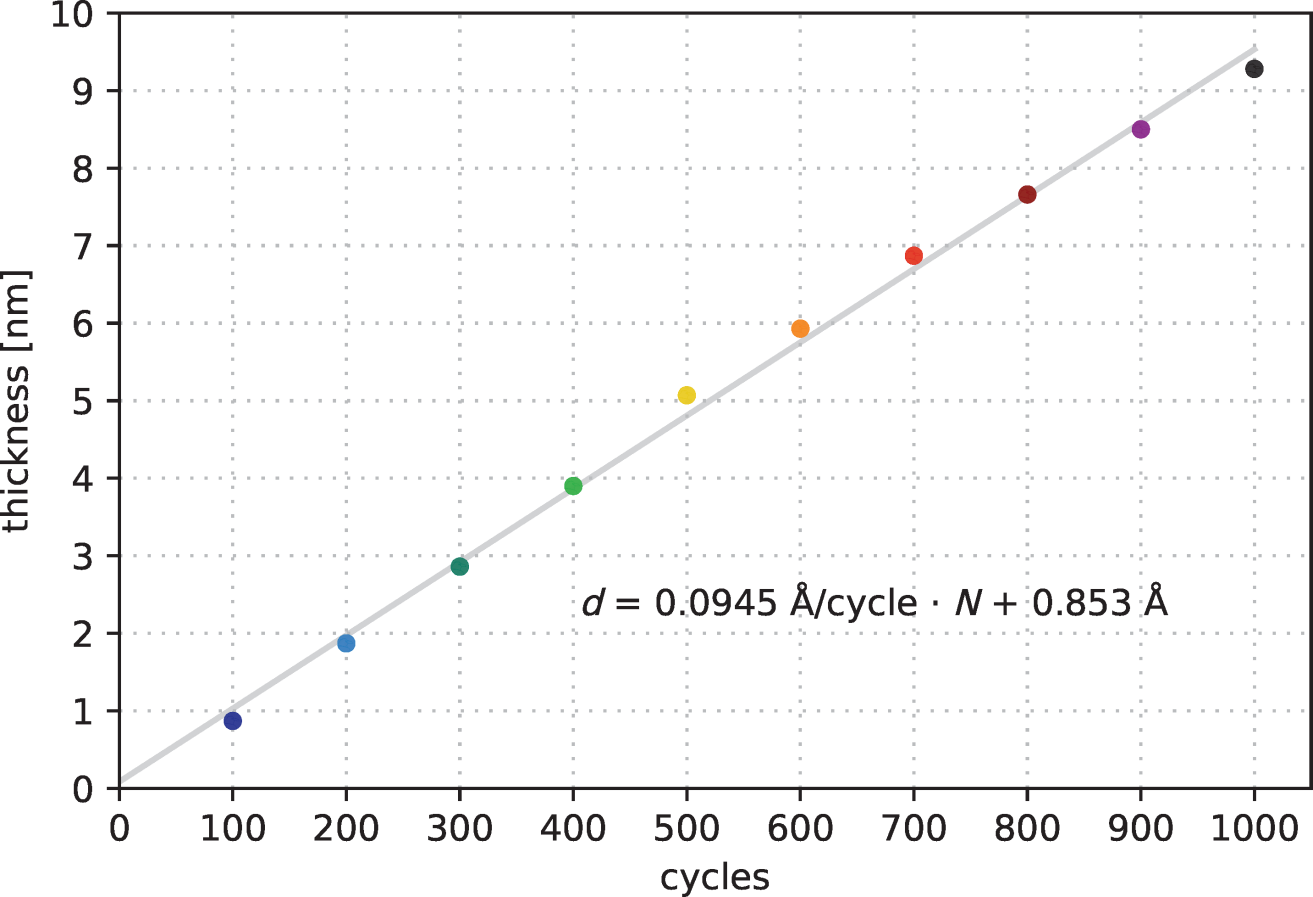
Film thickness evolution measured by in line spectroscopic ellipsometry for a deposition process at 85 °C.

The film thickness evolves linearly. Figure 7 shows the measured film thickness for a deposition process at 85 °C for subsets of 100 cycles. The corresponding ellipsometry raw data are shown in Supporting Information File 1, Figure S5. We assumed a linear approximation of type *d* = *r*·*N* + *d*_0_. This assumption comprises the film thickness *d,* the growth rate *r*, the number of cycles *N*, and an offset *d*_0_. The linear assumption for the process at 85 °C matches the measured data within the uncertainty range. The offset is almost negligible.

**Figure 8:**
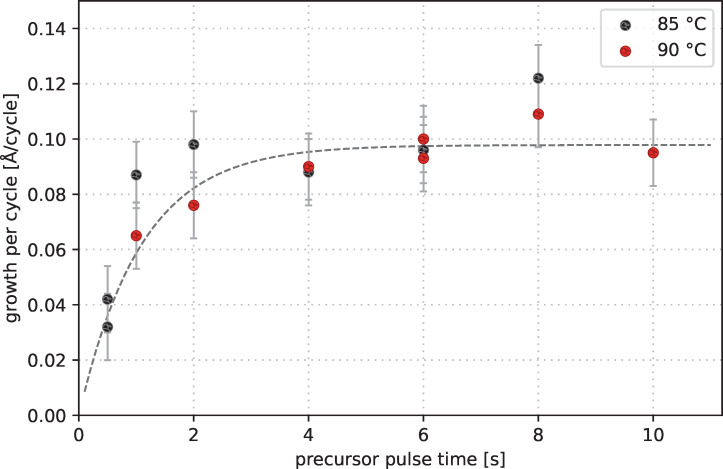
Influence of precursor pulse time on growth rate for depositions at 85 and 90 °C including a saturation curve of type *r* = *r*_0_·(1 − *e*^−^*^a^*^·^*^t^*) matching the 90 °C data points.

An important property of the deposition via ALD is the surface saturation. This means that the reactive sites of the substrate surface covered by the precursor will saturate with increasing duration of the precursor pulse resulting in an upper limit of the growth rate per cycle. This saturation follows an exponential decay curve [40]. Adapting Tuomo Suntola’s assumption of the surface occupation probability, we use a saturation curve of type *r* = *r*_0_·(1 − *e*^−^*^a^*^·^*^t^*), where *r* is the deposition rate in Å/cycle, *r*_0_ is the deposition rate at saturation, *a* is an arbitrary factor, and *t* is the precursor pulse time. Figure 8 shows the influence of the precursor pulse duration on the growth rate at 85 and 90 °C. The plotted saturation curve has been fitted to the data of 90 °C. The best matching parameters are *a* = 0.917 1/s and *r*_0_ = 0.098 Å/cycle. Using these parameters, a growth rate of 99% of the maximum growth rate is reached after 5.02 s precursor pulse time. Following these results, a precursor pulse time of 6 s has been chosen to reach the saturation state safely.

**Figure 9:**
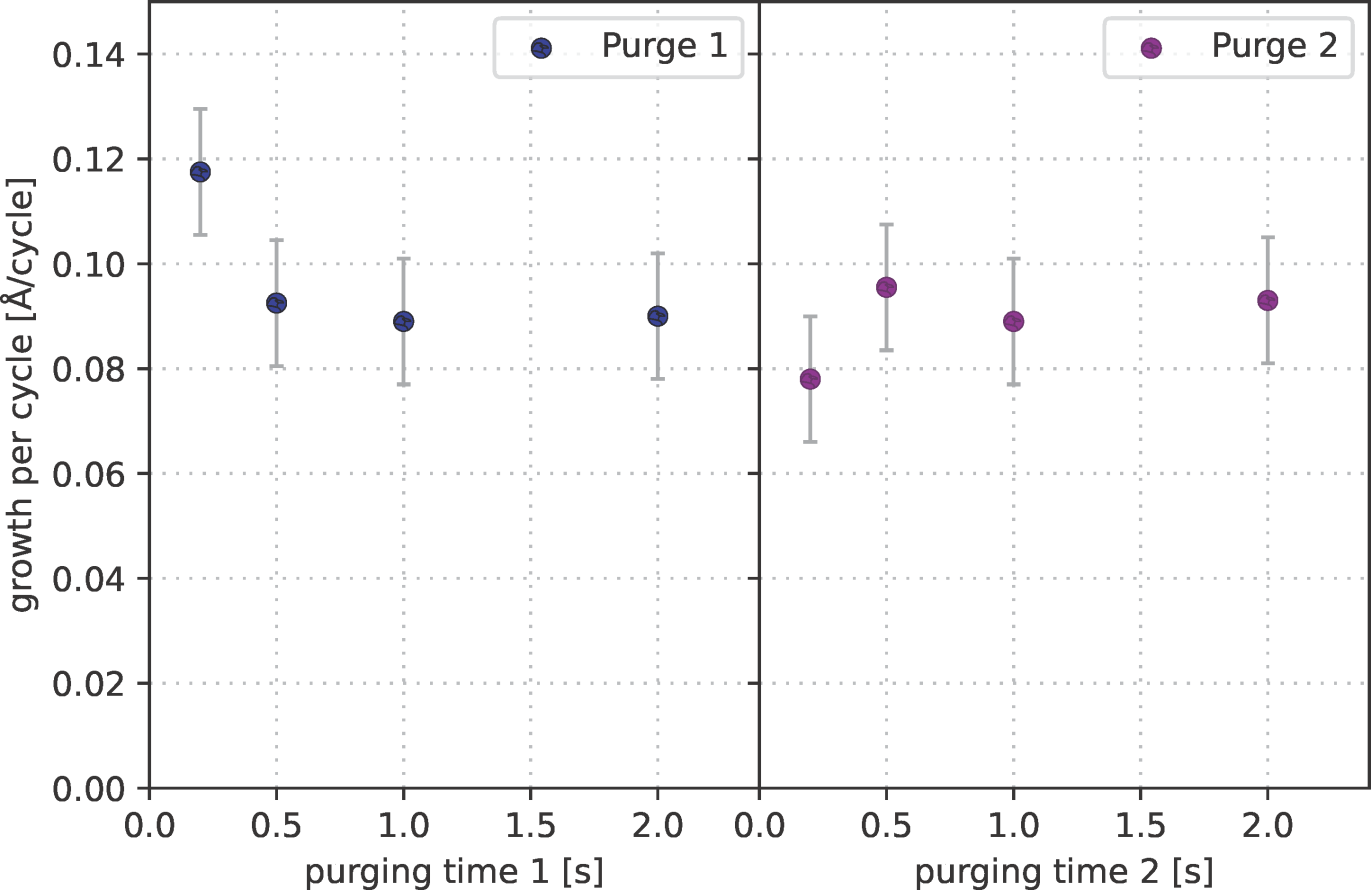
Influence of purging time after the precursor pulse (purging time 1) and after the H_2_ plasma pulse (purging time 2) on growth rate at 85 °C.

The purging time after the precursor pulse also may affect the deposition rate. Insufficient purging may result in an increased deposition rate as the remaining precursor can directly react in the gas phase within the hydrogen plasma. Figure 9 shows the influence of different purging times for ALD processes at 85 °C. The processes were done with a precursor pulse time of 6 s, which entails full surface saturation, as shown before. The growth rate is significantly increased for purging times below 0.5 s. With 0.2 s purging time, the growth rate rises to 0.118 Å/cycle. In contrast, the growth rate during the ALD process is nearly independent of the purging time after the hydrogen plasma pulse. For further investigations, a purging time of 1 s has been set for both purges.

**Figure 10:**
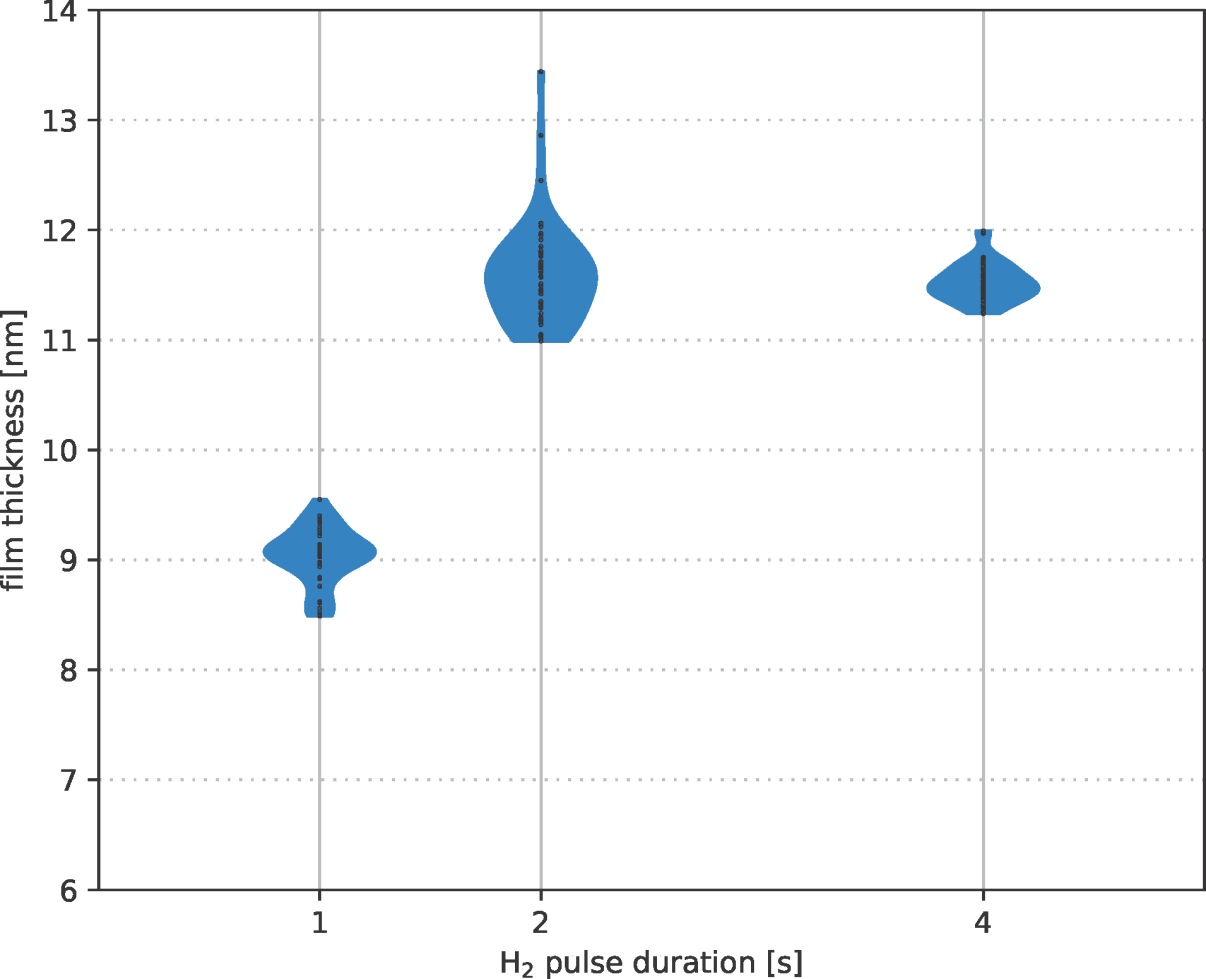
Influence of the H_2_ plasma pulse length on film thickness with the corresponding thickness distribution as violin plots for 85 °C processes with 1500 cycles and H_2_ pulse lengths of 1, 2, and 4 s, respectively.

**Figure 11:**
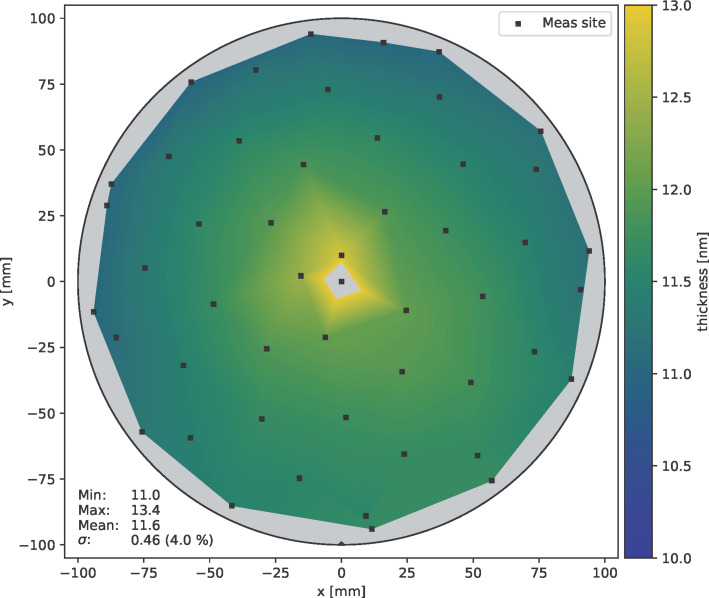
Thickness distribution of cobalt film on a 200 mm wafer after 1500 cycles at 85 °C with 2 s H_2_ plasma pulse; measured by ellipsometry.

**Figure 12:**
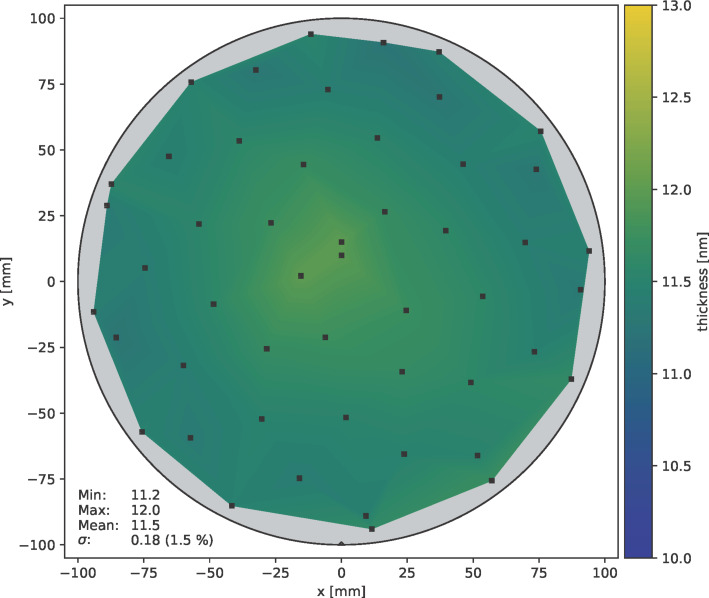
Thickness distribution of cobalt film on a 200 mm wafer after 1500 cycles at 85 °C with 4 s H_2_ plasma pulse; measured by ellipsometry.

The duration of the hydrogen plasma pulse also has a significant influence on the deposition behaviour. Figure 10 shows the thickness distribution of ALD layers deposited at 85 °C after 1500 cycles for different plasma pulse times as violin plot [41]. This plot shows the film thickness distribution on the wafer surface for each plasma pulse time. The results show that after 2 s H_2_ plasma pulse, the layer thickness reaches a maximum value independent of further increasing pulse times. With further increasing plasma pulse duration, the thickness variation over the wafer decreases from 4.0% (at 2 s) to 1.5% (at 4 s) relative standard deviation. The corresponding wafer maps of the thickness distribution are shown in Figure 11 (2 s H_2_ plasma) and Figure 12 (4 s H_2_ plasma), respectively.

**Figure 13:**
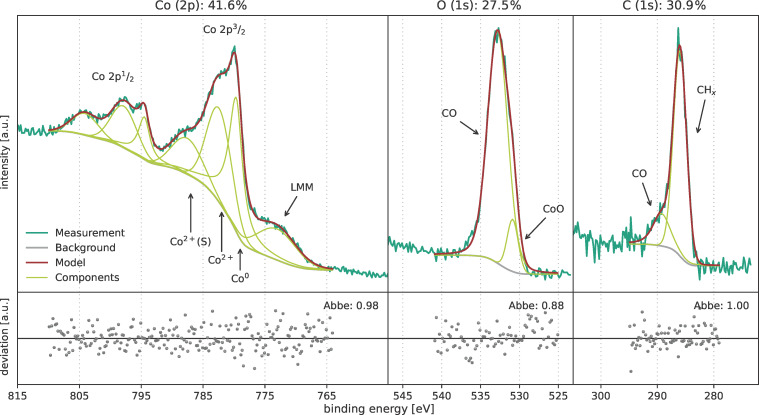
XPS results of a cobalt film deposited at 35 °C by ALD with [Co_2_(CO)_6_HC≡CC_5_H_11_] precursor.

The films from the optimised ALD processes were analysed by ex situ XPS measurements in order to determine the film compositions. As shown before, films deposited at 35 °C had a slightly increased growth rate. This indicates a different deposition mode, probably caused by incomplete precursor decomposition during the plasma pulse. The XPS measurement and the calculated composition of a film deposited at 35 °C are shown in Figure 13. The film is dominated by carbon and oxygen contaminations. The overview spectrum of this measurement is shown in Supporting Information File 1, Figure S3. The present peaks were deconvoluted analogously to the peaks of the CVD film (see Figure 5). The cobalt 2p peak consists of two features, namely a metallic peak (Co^0^ at 779.6 eV) and the two peaks of oxidised cobalt (Co^2+^ at 782.4 eV and Co^2+^(S) at 787.6 eV). Additionally, the spectrum consists of the L_3_M_23_M_45_ Auger transition peak (LMM at 773.1 eV) [42]. The oxygen peak consists of two parts, that is, a dominant Co−O bonding feature and a weaker CO feature. The carbon peak also consists of a weak CO feature and a dominant CH*_x_* part. These results show that a temperature of 35 °C does not provide sufficient thermal energy. In consequence, the precursor ligands are removed only to a minor extent.

**Figure 14:**
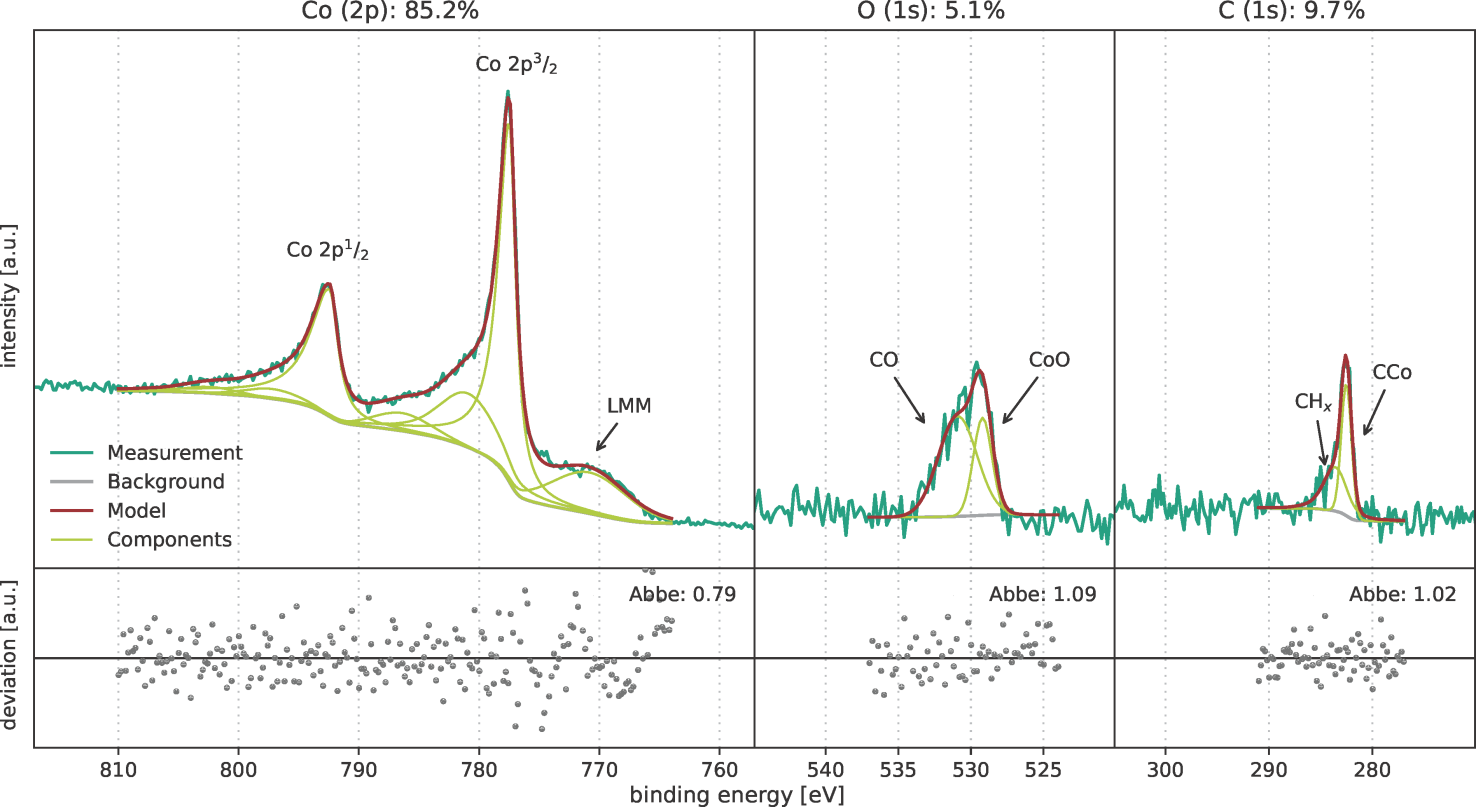
XPS measurements of a cobalt film deposited at 85 °C by ALD with [Co_2_(CO)_6_HC≡CC_5_H_11_] precursor.

The different films prepared in the determined ALD window from 50 to 110 °C exhibit comparable film compositions and differ significantly from the composition of the samples deposited at 35 °C (Figure 13) or 125 °C. Figure 14 shows the XPS results of a film deposited at 85 °C. An overview spectrum of this measurement is shown in Supporting Information File 1, Figure S4. The cobalt 2p doublet has the typical shape of cobalt in the metallic state, including two additional plasmon loss peaks (at 780.9 and 786.4 eV) and a LMM Auger transition peak at 770.8 eV [43]. The 2p^3^/_2_ peak maximum is located at 777.5 eV matching the reference value of Tan et al. [38] for metallic cobalt. This shows that the deposited film mainly consists of metallic cobalt. The film still contains contaminating elements, that is, 5.1 atom % of oxygen and 9.7 atom % of carbon. The O 1s peak consists of two features, namely one at 529.2 eV (CoO bond) and one at 530.9 eV (CO). The carbon 1s peak consists of two features. One feature is a small peak at 283.6 eV correlated to CH*_x_* bonds as shown in the previous XPS spectra (see Figure 5 and Figure 11). The second feature at 282.5 eV indicates the formation of cobalt carbide [35,44]. This also indicates a different deposition mode compared to the process at 35 °C. However, the sample was exposed to air before measuring XPS and this might be a source for contaminations. Future investigations will be done without breaking the vacuum prior to the XPS measurements.

## Conclusion

A plasma-enhanced ALD process for Co metal deposition based on [Co_2_(CO)_6_HC≡CC_5_H_11_] and H_2_ plasma was successfully developed. The process parameters were optimised regarding film homogeneity and required time. The respective ALD window in the temperature region from 50 to 110 °C was identified, which addresses also temperature-critical applications. The temperature-independent growth rate within this region was approximately 0.1 Å/cycle. The overall process optimisation concerning precursor pulse times, purge times, and plasma pulse time resulted in a homogeneous growth all over a 200 mm wafer. The XPS measurements show that within the ALD window cobalt is deposited in metallic state.

Further work will include the application of this process to high-aspect ratio structures. We will test the feasibility of the cobalt ALD film for direct electroplating of metallic copper. The film resistance has to be investigated and optimised as well.

## Supporting Information

File 1 Additional figures with XPS and ellipsometry raw data.
