# Supplementary material for: Low temperature atomic layer deposition of cobalt using dicobalt hexacarbonyl-1-heptyne as precursor
Source: Beilstein J Nanotechnol. 2023 Sep 15;14:951–63. doi: 10.3762/bjnano.14.78 (PMC10509559; doi:10.3762/bjnano.14.78)
Supplement: File 1 — Additional figures with XPS and ellipsometry raw data. [file Beilstein_J_Nanotechnol-14-951-s001.pdf]

BEILSTEIN JOURNAL OF NANOTECHNOLOGY

Supporting Information

for

Low temperature atomic layer deposition of cobalt using dicobalt hexacarbonyl-1-heptyne as precursor

Mathias Franz, Mahnaz Safian Jouzdani, Lysann Kaßner, Marcus Daniel, Frank Stahr and Stefan E. Schulz

Beilstein J. Nanotechnol. **2023,** *14,* 951–963. doi:10.3762/bjnano.14.78

Additional figures with XPS and ellipsometry raw data

License and Terms: This is a supporting information file under the terms of the Creative Commons Attribution License (https://creativecommons.org/licenses/by/4.0). Please note that the reuse, redistribution and reproduction in particular requires that the author(s) and source are credited and that individual graphics may be subject to special legal provisions.
The license is subject to the *Beilstein Journal of Nanotechnology* terms and conditions: (https://www.beilstein-journals.org/bjnano/terms)

Preliminary Experiments in CVD mode

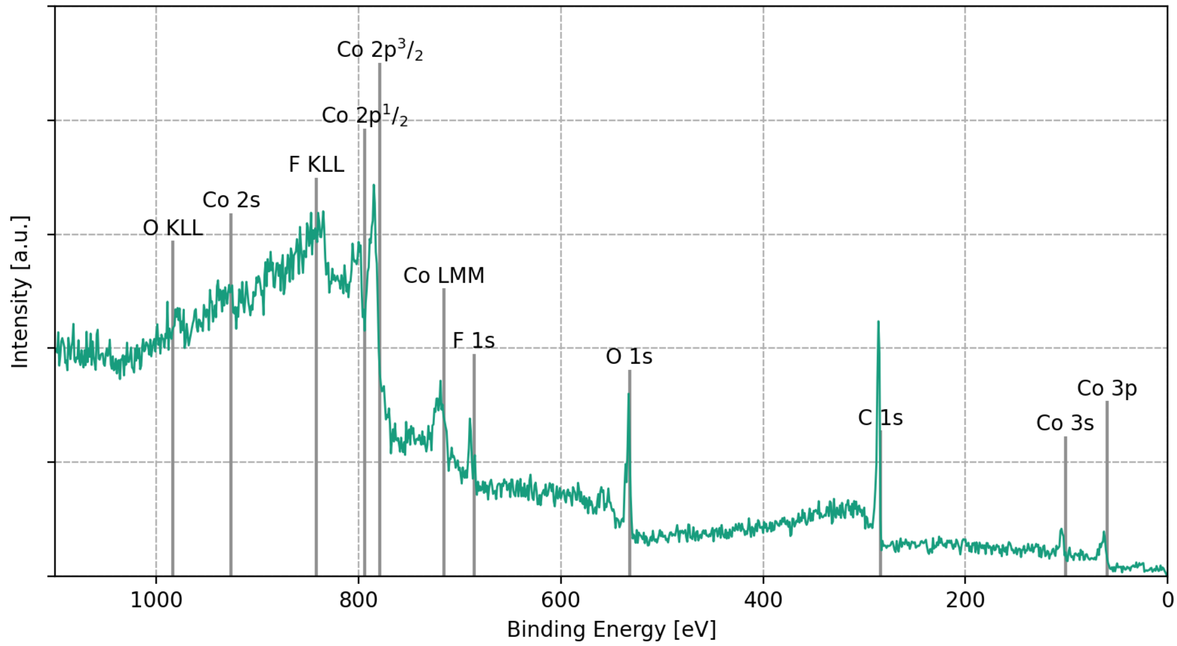

Figure S1: XPS overview spectrum of CVD film deposited at 150 °C and the relevant elemental binding energies and Auger transitions for the measured elements.

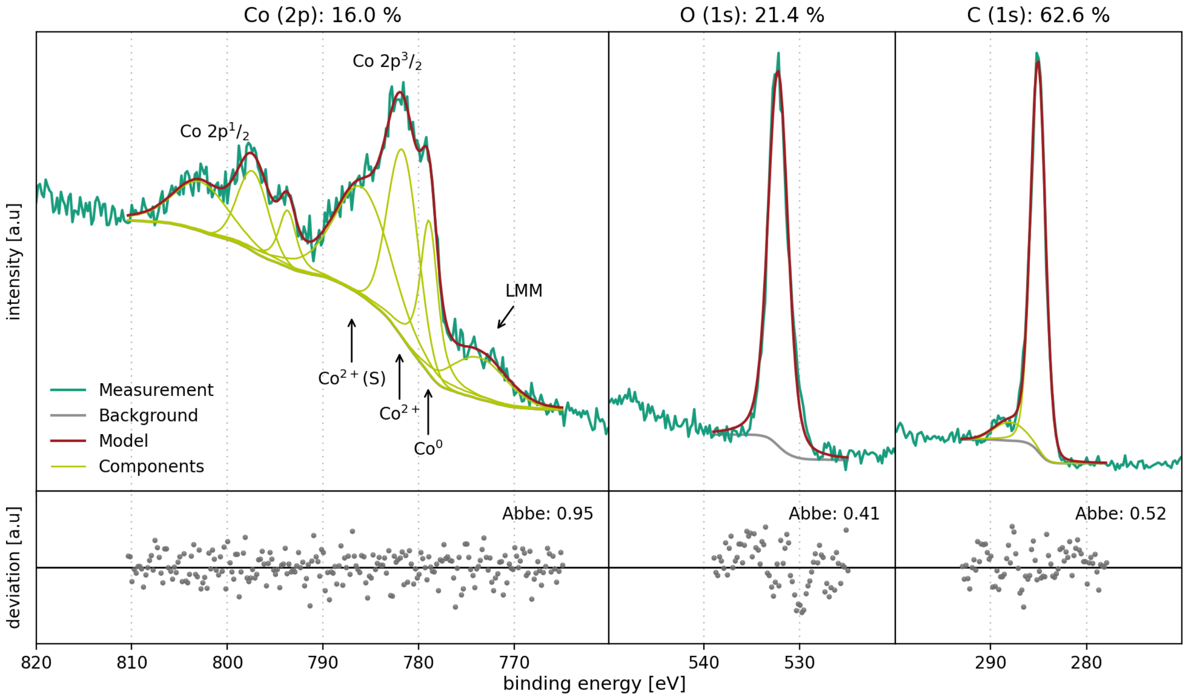

Figure S2: XPS measurements of CVD film deposited at 150 °C. The peaks for the O 1s and C 1s transitions were fitted with just one Voigt curve per main feature.

ALD with [$Co_2(CO)_6HC{\equiv}CC_5H_{11}$] and H_2 plasma

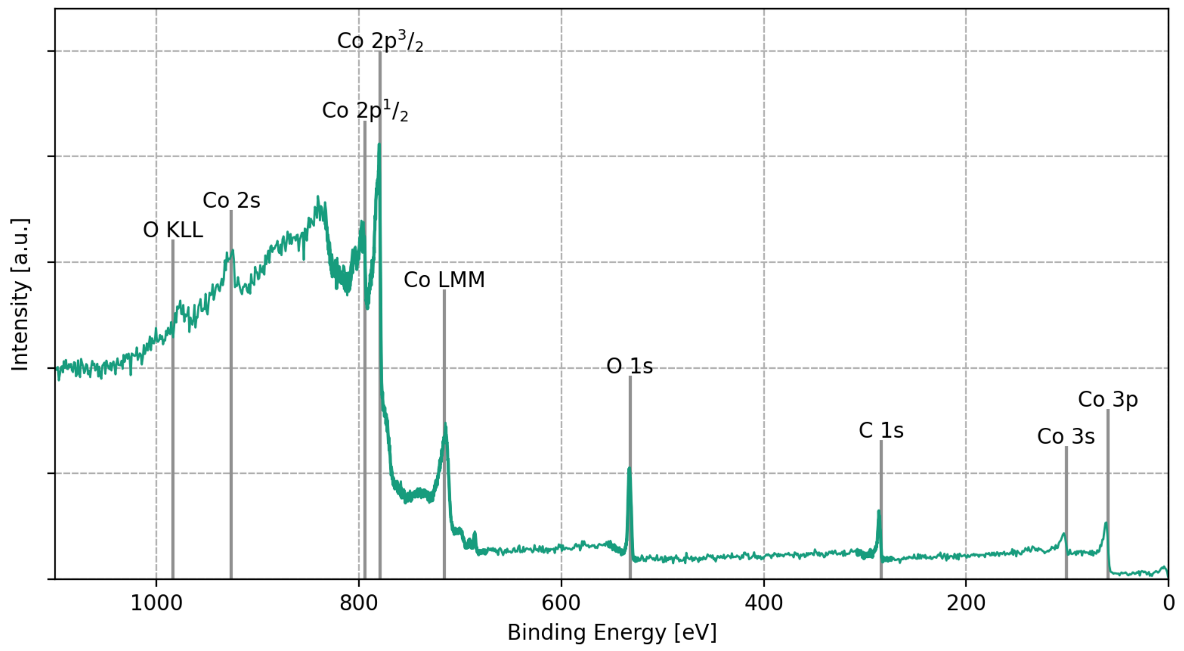

Figure S3: XPS overview spectrum of ALD film deposited at 35 °C and the relevant elemental binding energies and Auger transitions for the measured elements.

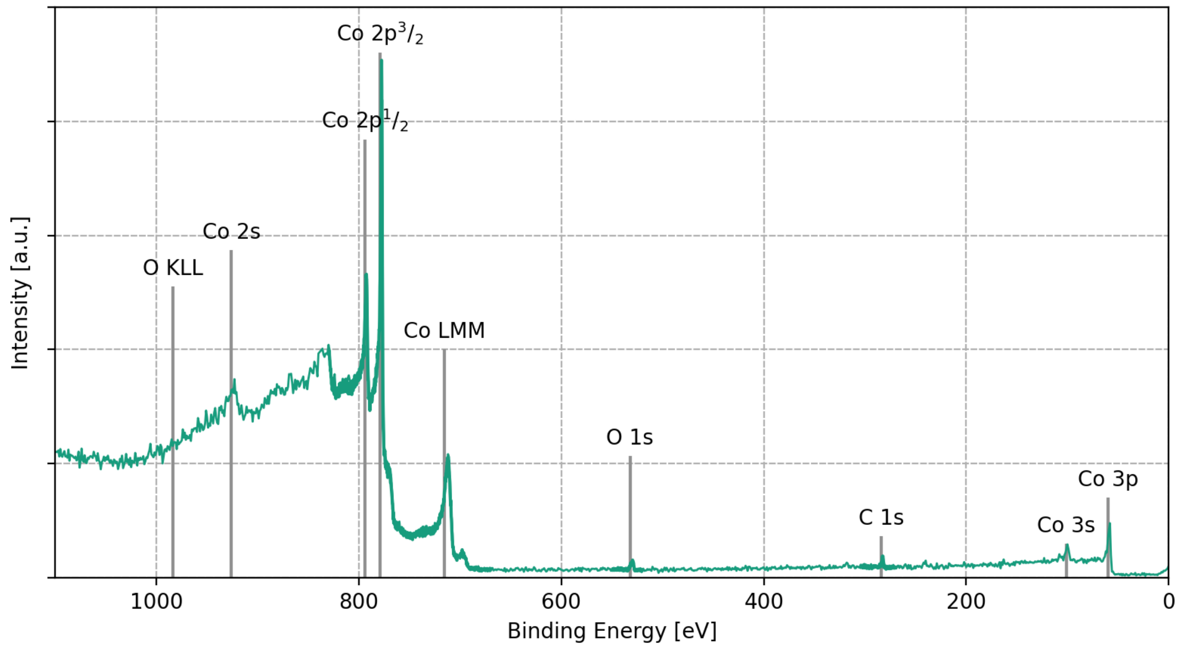

Figure S4: XPS overview spectrum of ALD film deposited at 85 °C and the relevant elemental binding energies and Auger transitions for the measured elements.

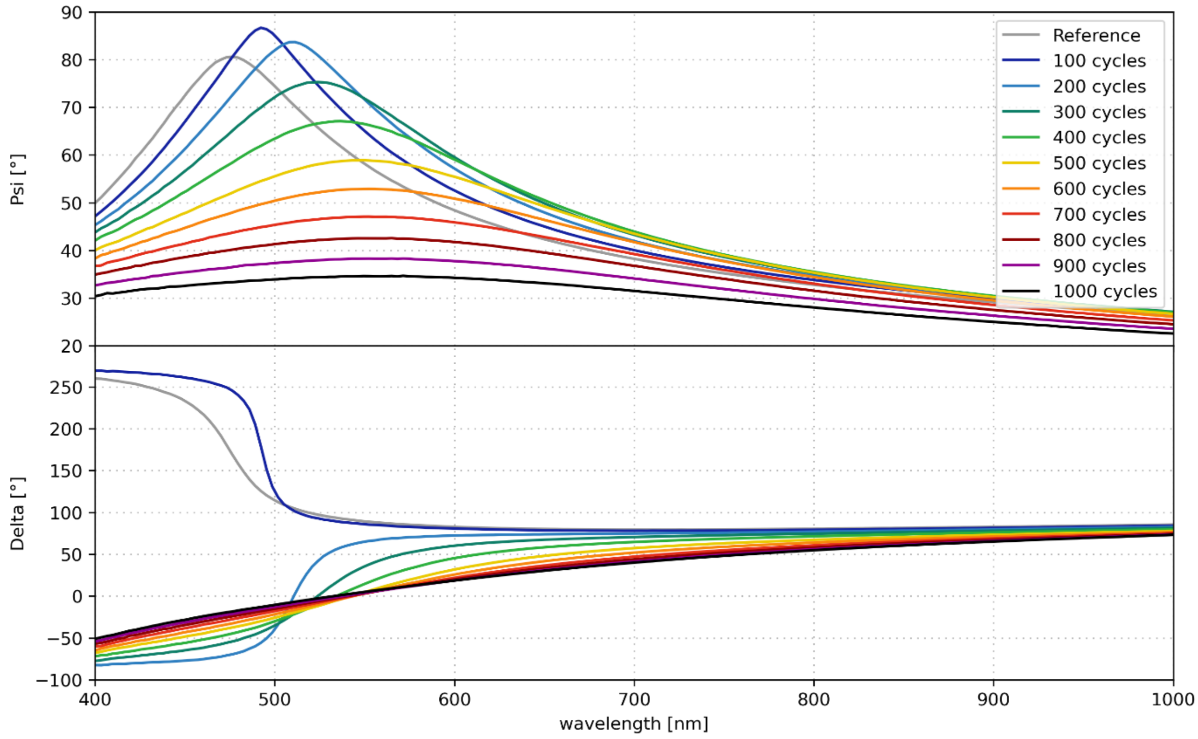

Figure S5: Ellipsometry raw data for ALD at 85 °C on 100 nm SiO_2.
